# Deep Learning Method Applied to Autonomous Image Diagnosis for Prick Test

**DOI:** 10.3390/life14101256

**Published:** 2024-10-02

**Authors:** Ramon Hernany Martins Gomes, Edson Luiz Pontes Perger, Lucas Hecker Vasques, Elaine Gagete, Rafael Plana Simões

**Affiliations:** 1 Department of Bioprocess and Biotechnology, School of Agriculture, São Paulo State University (UNESP), Avenue Universitária, 3780, Botucatu 18610-034, SP, Brazil; r.gomes@unesp.br (R.H.M.G.); hecker.vasques@unesp.br (L.H.V.); 2 Medical School, São Paulo State University (UNESP), Avenue Prof. Mário Rubens Guimarães Montenegro, s/n, Botucatu 18618-687, SP, Brazil; elpperger@gmail.com; 3 Dr. Elaine’s Clinic (Clínica Dra. Elaine), 398 Doutor Rodrigues do Lago, Botucatu 18602-091, SP, Brazil; doutoraelaine@gmail.com

**Keywords:** deep learning applied to diagnosis, prick test, measurement of wheal area, IgE response, sensitization to antigens

## Abstract

Background: The skin prick test (SPT) is used to diagnose sensitization to antigens. This study proposes a deep learning approach to infer wheal dimensions, aiming to reduce dependence on human interpretation. Methods: A dataset of SPT images (n = 5844) was used to infer a convolutional neural network for wheal segmentation (*ML model*). Three methods for inferring wheal dimensions were evaluated: the *ML model*; the standard protocol (*MA1*); and approximation of the area as an ellipse using diameters measured by an allergist (*MA2*). The results were compared with assisted image segmentation (*AIS*), the most accurate method. Bland–Altman analysis, distribution analyses, and correlation tests were applied to compare the methods. This study also compared the percentage deviation among these methods in determining the area of wheals with regular geometric shapes (n = 150) and with irregular shapes (n = 150). Results: The Bland–Altman analysis showed that the difference between methods was not correlated with the absolute area. The *ML model* achieved a segmentation accuracy of 85.88% and a strong correlation with the *AIS* method (ρ = 0.88), outperforming all other methods. Additionally, *MA1* showed significant error (13.44 ± 13.95%) for pseudopods. Conclusions: The *ML* protocol can potentially automate the reading of SPT, offering greater accuracy than the standard protocol.

## 1. Introduction

The skin prick test (SPT) is a relatively simple and easy-to-perform immediate reading test for immunoglobulin E (IgE)-mediated reactions, but its accuracy can be limited by the need for human interpretation [1,2]. The measurements of the wheal dimensions appearing on the skin after the puncture are made manually, which can cause different types of errors due to parallax, instrument resolution, and human error. In this way, the evaluation of the allergic reaction depends on the examiner [3]. Since the wheal area is proportional to the degree of sensitization (i.e., the stronger the immune response, the larger the wheal), this parameter is considered the main indicator for diagnosing an SPT [4,5]. A positive diagnosis is typically based on a wheal diameter 3 mm larger than the negative control and greater than half the diameter of the histamine response [6].

**Figure 1:**
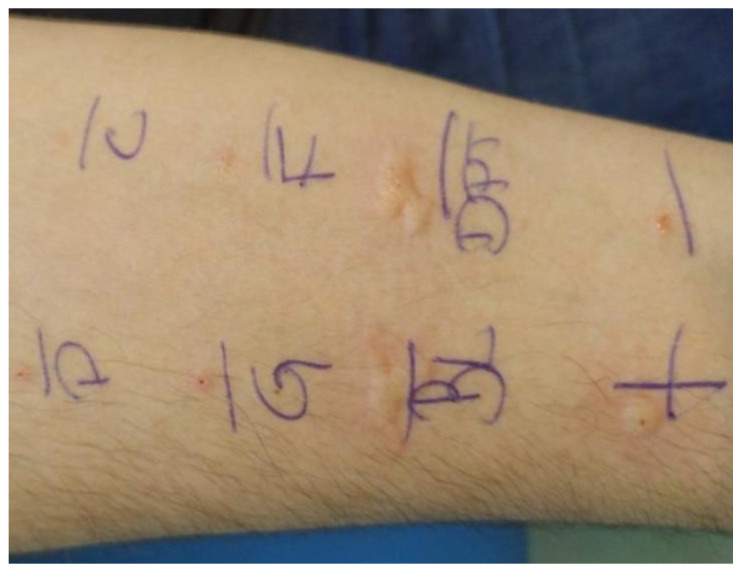
Prick test photography illustrating different wheal areas.

Subjectivity in wheal assessment arises from variations in its geometric contour, leading to inconsistent readings by different examiners or even by the same examiner at different times [3]. As illustrated in Figure 1, wheals can exhibit irregular geometric contours, further contributing to interpretation challenges [7]. Skin pigmentation presents another complication, making it difficult to identify the reaction outline [8]. Additionally, wheal location (e.g., volar vs. infrascapular region) can influence the result [9]. Therefore, standardized methods are necessary to address subjectivity in wheal assessment and improve diagnostic consistency.

In this regard, several research groups have been developing approaches using cameras, thermographic cameras, 2D or 3D scanners, and computer-aided color analysis for the automatic or semi-automatic detection of wheals formed in the SPT [7,8,10,11,12,13,14]. A notable advancement in this field is the Nexkin DSPT^®^, a commercial medical device designed to assist in detecting wheals and digitizing allergy skin test readings. This mechatronic system employs 3D laser technology to automatically locate wheals and measure their area and diameter [15]. However, it is important to note that, like the Nexkin DSPT^®^, most proposed approaches rely on specialized equipment, limiting their widespread adoption in clinical practice.

Automatic and semi-automatic methods for wheal detection, in addition to enhancing the objectivity of the SPT, can also lower testing costs. A study conducted a cost–consequence analysis to evaluate the financial implications of a computer vision-based SPT system (Nexkin DSPT) at *Inselspital*, a university hospital in Switzerland [16]. The findings indicated that the automated method saves an average of CHF 7 per test compared to manual reading. The main cost difference lies in human resources: the automated method requires only 3 min of personnel time at a cost of CHF 10.89, while the manual method takes 5.5 min and costs CHF 19.96. Overall, automated methods can significantly reduce reading time for SPTs, saving time for allergists and patients, while also leading to further financial savings. Moreover, most automatic methods for wheal detection significantly simplify SPT digitization, aiding in the creation of electronic medical records—a growing trend in healthcare systems [17].

Machine learning techniques and deep learning algorithms have emerged as powerful tools for image-based clinical diagnostics. Studies have shown that machine learning-generated classifier models outperform human evaluators in determining geometric parameters from images, such as area, radius, perimeter, and measures of shape complexity (compactness, smoothness, concavity) [18,19]. Semantic image segmentation is a machine learning technique utilizing deep learning algorithms, specifically convolutional neural networks (CNNs), and may be particularly suitable for identifying wheals in SPT images. It infers models to identify patterns in the image and classify pixels into predefined segments (or labels), allowing an accurate segmentation of the wheal area, which is a crucial requirement in SPT diagnosis [20].

Despite the great potential of CNN-based models, this technique has only recently been applied to diagnosing prick tests. In this regard, two works are worth mentioning: The first [21] uses a fringe projection system (consisting of two cameras, two lasers, and one projector) to acquire 3D images of the patient’s forearm, followed by a preprocessing step to remove the global surface, and finally utilizes a CNN to produce an output mask that detects wheals. The second [22] was recently published while we were preparing our manuscript. This study, similar to ours, proposes the use of CNNs for wheal segmentation, using only 2D images of wheals as input. However, the inference of this model was performed on a modestly sized dataset of 46 SPT images, which may explain the model’s relatively low sensitivity (56.21%).

The present work aimed to develop a deep learning-based computational protocol for applying image segmentation techniques to prick test photographs captured with conventional smartphones, intending to enhance the SPT reading method without the need for specialized equipment. Furthermore, we investigated whether the use of wheal area measurements, easily obtained with the segmentation model, can be a better indicator of the geometry of the reaction, and consequently of the sensitization. This approach has likely not been previously attempted due to limitations in earlier measurement instruments and has not been discussed in other studies.

## 2. Methods

### 2.1. Acquisition of Skin Prick Test Photos

SPT photos used to create the training and testing datasets were obtained from adult patients aged 18 to 65 years, of both sexes, who had not taken antihistamines or oral corticosteroids in the 10 days prior to the study. These patients were selected for convenience, adhering to all ethical and legal procedures involved in the research. The SPT was performed according to the established method by European Academy of Allergy and Clinical Immunology [6]. Briefly, the allergist conducted the prick test on the patients, who had previously signed the consent form. Immediately after 15 min of puncture application, (i) a square-shaped tag in chroma key color, referred to as the reference tag (RT), with known dimensions (3 cm × 3 cm), was placed on the forearm or upper back near the formed wheals; (ii) the smartphone was positioned parallel to the test region at a distance ranging from 10 cm to 30 cm; (iii) the focus was adjusted; and (iv) the photo was captured. Subsequently, the images were sent to the research group.

**Figure 2:**
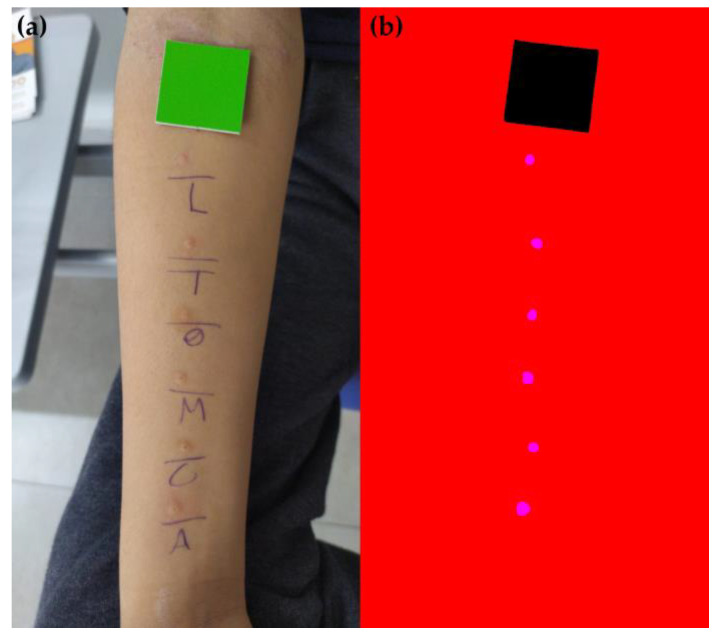
Illustration of (**a**) the original photograph and (**b**) the respective labeled image (manually annotated).

A total of 1461 photos were collected using smartphones of several brands, models, camera configurations, and operating systems. The image capture protocol did not include any prior calibration of pixel density or adjustments for resolution, focus, and lighting. The decision to avoid standardizing these parameters aimed to create a dataset with a wide diversity of instances (images), allowing the resulting model for wheal segmentation to be applied without requiring prior adjustments to these parameters. This approach simplifies the use of the proposed protocol for end users. Figure 2a illustrates a sample photo acquired using this method.

### 2.2. Dataset Standardization

The manual annotation of SPT photos was carried out using Corel Photo-Paint (version 23.1.0.389). Segments (or labels) were created in magenta (R = 255, G = 0, B = 255) for the pixels corresponding to the wheal area, in black (R = 0, G = 0, B = 0) for the pixels corresponding to the reference tag (RT), and in red (R = 255, G = 0, B = 0) for the remaining pixels in the image. Figure 2b shows an example of labeled SPT image. Subsequently, the ImageMagick software (version 7.1.1-38) [23] was used to resize the image and annotation pairs to 640 × 480 pixels in order to standardize the dataset and reduce the computational cost of image processing.

In machine learning models, overfitting occurs when a model becomes too specialized to the training data and performs poorly on unseen data. To enrich the dataset further and potentially reduce the overfitting of the deep learning model, a technique called data augmentation was employed using the ImageMagick software [24,25]. More specifically, geometric transformations were applied to the SPT images to make the deep learning model independent to changes in images position and orientation. For this purpose, each image underwent a sequential transformation process: (i) horizontal mirroring, (ii) vertical mirroring, and finally, (iii) another horizontal mirroring. This process effectively quadrupled the dataset size, resulting in 5844 images used for model training.

### 2.3. Machine Learning Model Training and Wheal Clustering

A generic and extensible fully convolutional neural (FCN) network developed by [20,26] was used to train the *ML model* for wheals segmentation. The network was implemented in Python using the TensorFlow library [27]. It begins with an input layer that accepts images of size 640 × 480 pixels with three color channels (RGB) and five convolutional blocks, identical to those proposed in the VGG-16 architecture [28], which serves as the encoder of the network. Each block is followed by max-pooling layers that progressively halve the spatial dimensions of the feature maps.

Following the encoding phase, two additional convolutional layers are introduced, featuring 4096 filters with sizes of 7 × 7 and 1 × 1, respectively. Subsequently, a 1 × 1 convolution is applied to incorporate class information, where the number of filters corresponds to the number of classes. The output of the last convolution proceeds to a 2× upsampling layer and is summed with the output of the fourth pooling layer, which was adjusted using a 1 × 1 convolution. The result of this operation is then upsampled again by a factor of 2 and similarly added to the output from the third pooling layer. Finally, the resulting output is upsampled by a factor of 8 and passed through a softmax layer, which converts the outputs into probabilities. All other layers use the ReLU activation function.

The model training was performed for 400 epochs with a batch size of 32 and a learning rate of 0.0001. The optimizer employed was Adam, and the loss function used was cross-entropy. The computational setup utilized for training included the following specifications: Processor: Intel^®^ Xeon^®^ E-2146G (6C/12T @ 3.50 GHz); RAM: 64 GB DDR4-2666; Segregated Operating System SSD: 960 GB SATA; Hard Disk: 2 TB SATA 6Gb/s; and GPU: nVidia^®^ GeForce^®^ GTX 1660 (1408 CUDA^®^ Cores, 6 GB).

Finally, a Python algorithm was developed using the Open Source Computer Vision Library (OpenCV) [29] to determine the area of each wheal after the segmentation. The algorithm first performs the pixel clustering of the instances classified as wheals by the *ML model*. Finally, the clustered pixels are used to estimate the individual wheal area using a simple proportion between the number of pixels of a wheal and the number of pixels of the RT, which has known area.

### 2.4. Evaluation of the Machine Learning Model Performance

An independent validation dataset consisting of 30 images containing 150 wheals was established to evaluate the performance of the proposed computational protocol. The images were obtained and segmented following the same procedures described in Section 2.1 and Section 2.2, with one key difference: a qualified allergist marked the contours of the wheal with a pen, which was used as a guide for the manual labeling of the images. This process, here referred to as assisted image segmentation (*AIS*), provided a reliable reference for assessing the *ML model* performance.

To assess the predictive performance of the ML model, we evaluated detection accuracy, segmentation accuracy, sensitivity, specificity, Dice similarity coefficient (DSC), and intersection over union (IoU). Detection accuracy was determined using Equation (1), where type I errors refer to the detection of non-existing wheals. The other metrics were evaluated from the variables of a confusion matrix derived from a cluster of pixels around the wheal (described in Section 2.3). The expected labels used in these analyses are those generated by the AIS method. The choice of cluster evaluation was made to specifically assess the predictive performance metrics in the segmentation of individual wheals.

(1) $$Detection\;accuracy\;\left(\%\right)=\frac{Correct\;detection}{Expected\;detection+Type\;I\;errors}\times 100$$

The agreement between the inferred model (*ML*) and the standard medical method (largest diameter of the wheal, *Medical LD*, and its perpendicular diameter, *Medical PD*, as measured by a professional) was evaluated based on the concordance analysis proposed by Bland and Altman [30]. We estimated the wheal area values from *Medical LD* and *Medical PD* using Equations (2) and (3) to compare the methods. Specifically, *MA1* represents the area of a circle with a diameter equal to the mean of *Medical LD* and *Medical PD*, while *MA2* represents the area of an ellipse with the major axis equal to *Medical LD* and the minor axis equal to *Medical PD*. To verify whether the sample size of the validation set was representative, we performed 15 data shuffles and analyzed the behavior of the cumulative mean and standard error. Paired Wilcoxon signed rank tests [31] and correlation tests were performed between the area values obtained by each method. Finally, the cumulative distribution function was determined for the areas estimated using the four methods (*ML*, *AIS*, *MA1*, and *MA2*).

(2) $$MA1\left({cm}^{2}\right)=\pi \times {\left(\frac{(Medical\;LD\;(cm)+Medical\;PD\;(cm))/2\;}{2}\right)}^{2}$$

(3) $$MA2\left({cm}^{2}\right)=\pi \times \frac{Medical\;LD\;(cm)}{2}\times \frac{Medical\;PD\;(cm)}{2}$$

All these analyses were performed using the R software (version 4.2.2, R Foundation for Statistical Computing, Vienna, Austria) [32] and Python3 (version 3.9, Python Software Foundation) [33].

### 2.5. Comparison of Area Estimates for Regular and Irregular Geometric Shapes Using Different Methods

**Figure 3:**
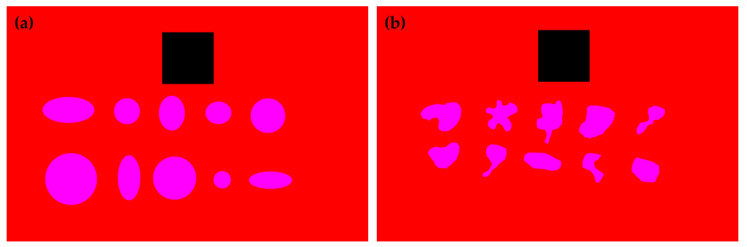
Sample of a segmented image containing only (**a**) shapes with regular geometric contour; (**b**) shapes with irregular geometric contour. The shapes are colored magenta and the RT is colored black.

Previous studies have demonstrated that computationally assisted methods can deviate from the standard protocol for measuring wheal dimensions, particularly when dealing with irregularly shaped wheals [34]. To evaluate these potential divergences, two distinct groups of segmented images were created. The first group (Group 1—Regular Shapes) consists solely of shapes with regular outlines, which can be approximated by circles or ellipses. The second group (Group 2—Irregular Shapes) comprised exclusively shapes with highly irregular geometric contours. Figure 3 presents a sample of each group here described. In all images, a square with known dimensions (3 cm × 3 cm), similar to the RT, was inserted to provide a scale for determining the shapes’ diameters and areas.

The image files from Group 1—Regular Shapes and Group 2—Irregular Shapes contain 10 different segmented shapes (as shown in Figure 3). Each group comprised a total of 15 images files, resulting in 150 shapes for Group 1 and 150 shapes for Group 2. The shape pixels in each image were clustered using the protocol proposed in this study (see Section 2.3). Additionally, the largest diameter (*Computational LD*) and diameter perpendicular to the largest diameter (*Computational PD*) were computationally determined. Using these parameters, the shape areas were also estimated as the area of a circle with a diameter equal to the average of computational LD and computational PD (similar to Equation (2)), and as an ellipse with the major axis equal to computational LD and the minor axis equal to computational PD (similar to Equation (3)). We also assessed the area of the figures using the function “pixel distribution histogram”, available in Corel Photo-Paint software, here referred to as *Corel Area*. The percentage deviation between the methods was calculated and used to evaluate their performance in predicting the areas of shapes with regular and irregular geometric contours.

## 3. Results

**Figure 4:**
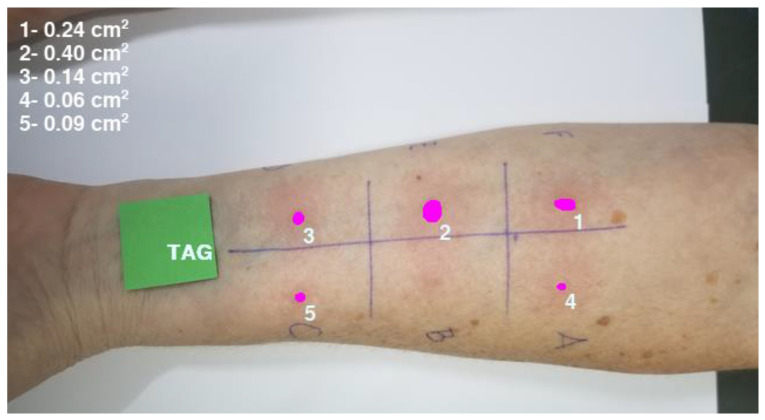
An example of an image containing the segmented wheals identified by a number and a report in the upper left subtitle containing the wheal areas in cm^2^.

The proposed *ML* protocol was able to segment and cluster wheals in SPT images. The area of each wheal was estimated using a simple proportion between the number of pixels from RT (with a known area) and from the segmented wheal. The *ML* protocol generated an image containing the segmented wheals, each identified with a number, accompanied by a report in the upper left corner summarizing the wheal areas in cm^2^. Figure 4 shows an example of an SPT image segmentation performed by the *ML* protocol.

**Figure 5:**
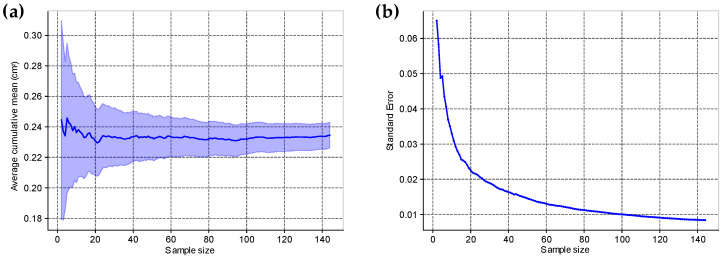
(**a**) Average curve of the cumulative mean of the areas determined by the ML model. The shaded area represents the region between the cumulative mean ± standard error. (**b**) shows the decay of the standard error as a function of sample size.

The *ML model* for wheals segmentation had its performance measured on a validation dataset with 30 different images and 150 wheals. In this dataset, nine wheals were not correctly detected, with three detections of non-existing wheals (type I error) and six wheals that existed but were not detected (type II error), which resulted in a *Detection accuracy* of 94.12%. In the 144 correctly detected wheals, the segmentation model on average obtained a segmentation accuracy of 85.88%, a sensitivity of 70.29%, a specificity of 98.94%, a DSC of 80.85%, and an IoU of 69.65%. Figure 5 illustrates the average behavior of the cumulative mean of the areas determined by the *ML model*, along with the standard error. The number of wheals in this dataset clearly resulted in the stabilization of the mean and the minimization of the standard error, demonstrating that it is representative to evaluate the predictive performance of the model.

**Figure 6:**
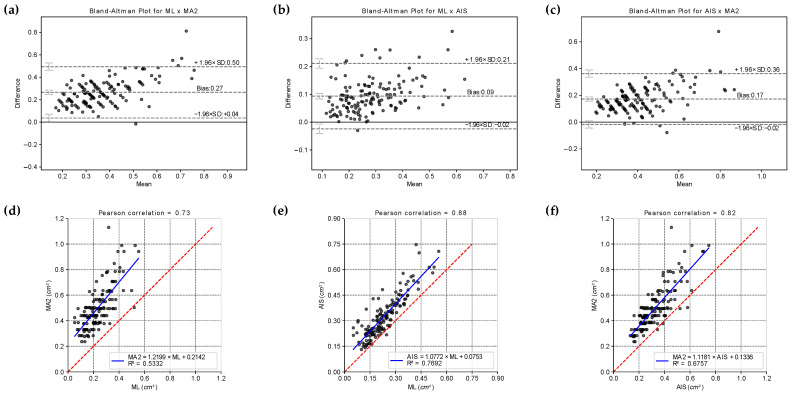
Bland–Altman analyses for (**a**) *ML* × *MA2*, (**b**) *ML* × *AIS*, and (**c**) *MA2* × *AIS*. Correlation plots between (**d**) *ML* and *MA2*, (**e**) *ML* and *AIS*, and (**f**) *MA2* and *AIS*. It should be noted that the slope of the lines is close to 1.0, while the intercepts are close to the Bland–Altman bias value, especially in the line relating the *AIS* and *ML* methods. The results for *MA1* and *MA2* are similar and, for simplicity, the graphs for *MA1* are not presented in this figure.

The paired Wilcoxon signed rank test showed a statistically significant difference between the areas *MA1*, *MA2*, *AIS*, and *ML* (*p*-value < 0.001 in all tests). Despite this result, the Bland–Altman analyses (Figure 6) indicated that the difference between the measurement methods is concentrated around a bias (mean difference), indicating no correlation between the difference obtained between the measurement methods and the magnitude of the variable area.

**Figure 7:**
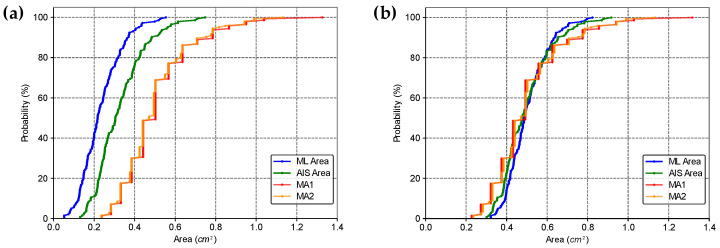
Cumulative distribution functions for the areas estimated by the four methods for (**a**) the original data, and (**b**) adding the bias between *ML*, *AIS*, *MA1*, and *MA2*.

We also determined the cumulative distribution function of the areas estimated by the four methods, *ML*, *AIS*, *MA1*, and *MA2* (Figure 7). This analysis confirmed the statistically significant difference observed in the paired Wilcoxon signed rank test, and clarified that the distributions can be superimposed by shifting the curves in the bias value. Thus, biases can be used to relate the areas obtained by different methods. Furthermore, the different methods can also be related using linear equations.

It can be noted in Figure 6 and Figure 7 that there is a uniform pattern in the scatter of points in all analysis performed for *MA1* or *MA2*, and it should be clarified that this does not represent a bias in the dataset. This pattern occurred because the *MA1* and *MA2* were determined using a measuring instrument with precision of 1 mm. As a result, the graphical representation of these values (or the direct analyses performed with them) tends to show a pattern of points discretization. However, this pattern was not observed for the *ML* and *AIS* methods. This difference can be explained by the fact that the computational variables used to determine the area of the wheals were declared as floating types (i.e., real numbers), which have a precision of 10^−8^. This precision is relatively high, so the graphs for the results from *ML* and *AIS* methods did not show point discretization. This consideration is important because it highlights that the conventional method of determining wheal dimensions has an accuracy limited by the precision of the measuring instrument used for the SPT (not to mention other possible sources of error). In contrast, the computational method performs a pixel-by-pixel analysis, in which the accuracy is limited by the image resolution. Generally, any current smartphone can capture images with sufficient resolution to surpass the accuracy obtained using the *MA1* and *MA2* methods.

**Figure 8:**
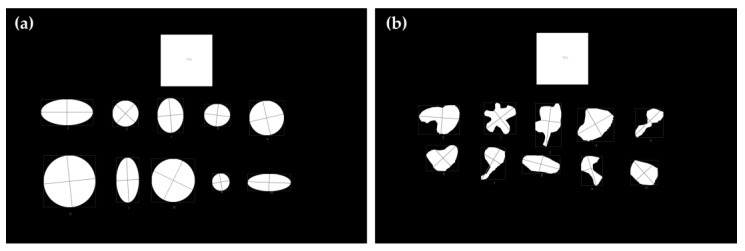
*Computational LD* and *Computational PD* determined for the (**a**) regular and (**b**) irregular geometric shapes of the segmented images presented in Figure 3. This figure demonstrates that the computationally determined diameters serve as reliable estimates of *LD* and *PD*.

The following results show the performance of the methods evaluated in this study to determine the *LD*, *PD*, and area of shapes with regular geometric contours (circles or ellipses) and irregular geometric contours (corresponding to pseudopods). The proposed method for determining *Computational LD* and *Computational PD* values yielded results that closely matched the mathematically expected *LD* and *PD* values for the regular images. Figure 8 illustrates an example of an image from each group (regular shapes and irregular shapes) where *Computational LD* and *Computational PD* were calculated. For the regular shapes in Figure 8a, the mean percentage deviations of *Computational LD* and *Computational PD* were 0.3754% and 0.5410%, respectively. The minor deviations observed in the regular shapes are likely due to the fact that the images represent circles and ellipses rather than ideal mathematical objects. Since our computational method for determining the diameter is invariant to the geometric shape and relies solely on the estimate of the geometric center (easily determined by averaging the coordinates of the shape), we believe that this method provides accurate estimates of *Computational LD* and *Computational PD* for the irregular dataset. This accuracy is further confirmed visually.

**Figure 9:**
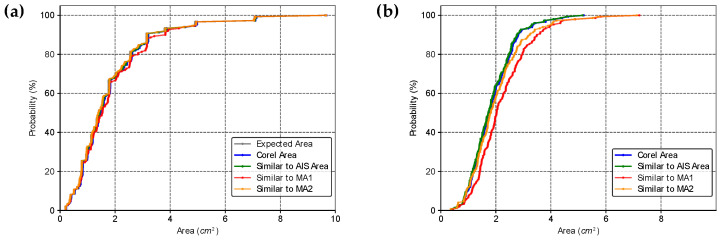
Cumulative distribution functions for the areas estimated by different methods. In (**a**) Group 1—Regular shapes; in (**b**) Group 2—Irregular shapes. The expected area was calculated from the axes defined to create the ellipses and circles, the *Corel Area* was determined using the pixel distribution histogram function available in the Corel Photo-Paint software, and protocols similar to the *AIS* area, *MA1*, and *MA2* were calculated similarly to the *AIS* area (or also to the *ML* area), *MA1*, and *MA2*, respectively. Note that for areas similar to *MA1* and similar to *MA2*, the computationally determined diameter was used.

**Figure 10:**
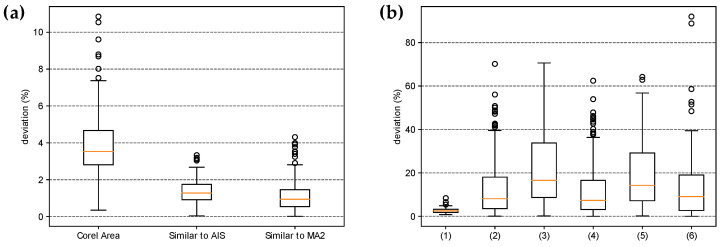
Boxplot for percentage deviations of area values between different methods for: (**a**) Group 1—Regular shapes and (**b**) Group 2—Irregular shapes. In (**a**), the values obtained by the different methods were compared with the expected area. The boxplot for the area similar to *MA1* was omitted for clarity; its minimum, mean, and maximum values were 0.02%, 5.65% ± 13.04%, and 96.10%, respectively. In (**b**), the boxplot names were coded for better illustration, and their meanings are as follows: (1) similar to *AIS* × *Corel Area*; (2) similar to *AIS* × similar to *MA2*; (3) similar to *AIS* × similar to *MA1*; (4) *Corel Area* × similar to *MA2*; (5) *Corel Area* × similar to *MA1*; and (6) similar to *MA2* × similar to *MA1*.

The *LD* and *PD* are essential parameters for evaluating the accuracy of the standard medical protocol used to infer wheal dimensions. *MA1* assumes that the wheal contour can be approximated as a circle with a diameter equal to the average of *LD* and *PD*, while *MA2* assumes the wheal has an elliptical shape, with the major axis corresponding to *LD* and the minor axis corresponding to *PD*. Thus, the area of the wheal can be estimated as described in Equations (2) and (3). However, in this study, we propose that approximating the wheal area as an ellipse or circle may be inaccurate, particularly for wheals with irregular contours. Instead, we suggest that using a simple proportion between the pixel count from RT and the pixel count from the wheal may provide more accurate results, regardless of the shape of the wheal. To support the proposed statement, the results for two distinct groups of shapes are presented below: Group 1—Regular shapes and Group 2—Irregular shapes. The cumulative distribution of the area values estimated by the different methods and the boxplots of the percentage deviation values between the methods were determined for both groups, and the analyses are shown in Figure 9 and Figure 10.

In Group 1—Regular Shapes, the mean percentage deviations of the *Corel Area*, the protocol similar to *AIS*, and the protocol similar to *MA2* values relative to the expected area were 3.82% ± 1.97%, 1.38% ± 0.64%, and 1.18% ± 0.94%, respectively. It is noteworthy that although the mean deviation of values calculated similarly to *AIS* is slightly higher than that calculated similarly to *MA2*, the maximum deviation for values similar to *MA2* is greater (4.31% compared to 3.33%). Overall, all methods performed well in estimating the area of regular shapes; among them, the least effective methods were *Corel Area* and the protocol similar to *MA1*.

The area calculation method similar to *AIS* (simple proportion using an RT) is invariant to the geometric shape of the figure and showed low deviations from the expected areas in Group 1—Regular Shapes. Therefore, we used this method as a reference for analyzing irregular figures, as we had no mathematical means of determining their expected areas. In Figure 9b, it can be seen that the order of adherence to the area values calculated similarly to *AIS* was in decreasing order: *Corel Area*, Similar to *MA2*, and Similar to *MA1* (Boxplots (1), (2), and (3)). This result is consistent because, although the areas calculated using the Corel Photo-Paint pixel distribution histogram function exhibited larger deviations than methods similar to *AIS* and similar to *MA2* in regular figures, this method is also invariant to geometric shape and seems to have maintained the magnitude of its deviations relative to values calculated similarly to *AIS*. In contrast, *MA1* and *MA2* are approximations highly dependent on the geometric shape of the figure, resulting in larger deviations from the expected area values. What should be emphasized in these results is that for figures with irregular contours, the standard medical protocol can lead to large deviations from the expected area values, thus compromising the quality of the diagnosis.

## 4. Discussion

**Figure 11:**
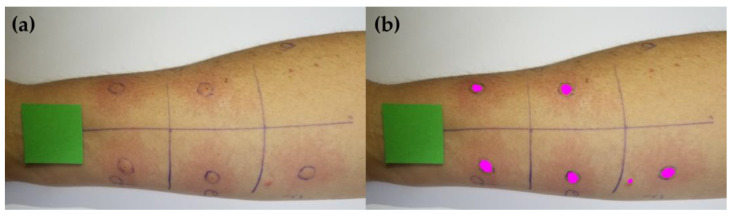
(**a**) Patient’s arm 15 min after the punctures. (**b**) *ML model* segmentation where a type I error was identified.

Regarding the three instances that presented type I errors in the validation dataset, it was observed that two puncture marks in the negative control were detected as wheals. The third type I error is shown in Figure 11 and refers to a small reddish mole on the patient’s arm that was segmented by the *ML model*. The light conditions used to take the picture, the patient’s skin color, or the small size of the wheal were possible causes of type II errors. Although the detection accuracy was considered satisfactory, both error types tend to decrease with the increment of the number of instances (images) in the training dataset, which can be performed and evaluated in future studies. In addition, for clinical uses of the *ML* approach, it is expected that the wheal segmentation performed by the *ML model* will always be interpreted and validated by a health professional since the developed protocol aims to provide a tool to support humans to perform a faster and more accurate diagnosis, and not replace them.

The cumulative distribution functions confirm that the *ML* method produces measures closer to the *AIS* distribution than the *MA1* and *MA2*. Moreover, the values obtained by the *ML* method have a more continuous distribution than those obtained by the Medical Diameters, which may indicate a higher analysis resolution. This characteristic probably occurred because the Medical Diameters has its resolution linked to the graduation of the instrument used to measure the wheal diameter (usually a ruler or caliper). In contrast, the *ML* and *AIS* methods perform a pixel-by-pixel analysis.

The Pearson correlation coefficients between the measurement methods are shown in Figure 6. A strong correlation was found between the areas inferred through the *ML* and *AIS* methods (ρ = 0.88), which was considerably more significant than the correlation between the *AIS* and *MA1* (ρ = 0.80) and *MA2* (ρ = 0.82) and was consistent with the results of Bland–Altman’s analysis. In previous studies carried out by our group [34], we determined a statistically significant Pearson correlation coefficient between skin temperature variation in the wheal (during the SPT) and the area determined by a similar *AIS* method. Since the *ML* method had a strong correlation with the *AIS* method, it is reasonable to assume that the *ML* method is also associated with the wheal temperature during the skin sensitization reaction, which is a characteristic known to be proportional to the intensity of sensitivity to the antigen.

Some patients with high levels of sensitization to an antigen may be more susceptible to developing wheals with irregular contours and the formation of pseudopods [35]. In these cases, approaches that use medical diameters to infer sensitization may not be the most appropriate. These methods approximate wheal areas using only the values of *LD* and *PD* (or by calculating the mean of both diameters) [4,6]. In other words, the method for determining the area of the wheals approximates the sensitization area by assuming an elliptical shape, in which the major axis corresponding to *LD* and the minor axis to *PD*, or, alternatively, by using a circle with a diameter equal to the average of *LD* and *PD*. However, for irregular shapes, such as pseudopods, this approximation may differ significantly from the real area of the wheals, as our results suggest. Moreover, the standard protocol to infer skin sensitization can be associated with other problems, such as parallax error, which may further reduce diagnostic accuracy.

It should be highlighted that the standard medical protocol for inferring the area of skin sensitization was developed based on the measurement tools that were available and feasible at the time the protocol was defined. These instruments are essentially tools for measuring linear dimensions, such as rulers or calipers. However, our results, along with findings from other studies, indicate that new technologies can be used to more accurately determine the area of skin sensitization. All of this points to the need to review and update the standard prick test protocol to improve its diagnostic accuracy.

Our model was inferred using an FCN architecture for image segmentation. This uses the VGG-16 as an encoder and is a classic network architecture introduced by [20]. It has been extensively used in semantic image segmentation studies. Currently, new architectures have been developed and applied to this type of problem, with U-Net being particularly prominent [36]. U-Net has shown good performance in medical imaging studies [37]. In this context, future studies could benchmark our current FCN with U-Net architectures applied to the problem of wheals segmentation in SPT. Furthermore, we believe that our model has achieved sufficient performance to assist in the annotation of new images, thereby facilitating the expansion of the training dataset for future studies.

In this study, we propose that segmentation techniques using deep learning or other computationally assisted methods can be applied to determine the wheal areas. These techniques can be fully automated, making the prick test diagnosis entirely objective and independent of the professional’s experience or interpretation when measuring the wheal dimensions. The need for manual measurement using tools such as rulers or calipers is eliminated, saving time for both the professional and the patient. The proposed methodology can also expedite patient care, potentially leading to significant cost savings for healthcare services performing the SPT. Furthermore, it can be easily integrated into hospital information systems, facilitating the generation of reports and medical records that include SPT images and patient history.

## 5. Conclusions

The results indicate that the measurements obtained from the developed ML method were consistent with those from the other evaluated methods (AIS and Medical Diameters). Specifically, for figures with irregular contours, the proposed method for calculating wheal area offers more accurate estimates compared to those based on medical diameters. This is because the simple proportion between the RT pixel count and the wheal pixel count does not rely on a predefined shape (circle or ellipse), making it invariant to the geometric contour of the wheal. These findings suggest that the proposed protocol has the potential to automate the assessment of the SPT reaction and can be used in clinical practice. This would render the prick test diagnosis entirely objective, free from dependence on the professional’s experience or interpretation when measuring wheal dimensions. However, further extensive studies are necessary, primarily to standardize the use of area values for diagnosing antigen sensitization during SPT. Overall, these results underscore the need to reevaluate the prick test protocol in light of the new technological tools currently available.

## 6. Patents

The method presented in this paper resulted in a computer program registered at Brazil’s National Institute of Industrial Property (INPI) (Process No.: BR512021000570-8).

## Data Availability

The data presented in this study are available upon request to the corresponding author(s). (The data are not publicly available due to privacy and ethical constraints).
